# Impact of Aquatic Exercise on Respiratory Outcomes and Functional Activities in Children with Neuromuscular Disorders: Findings from an Open-Label and Prospective Preliminary Pilot Study

**DOI:** 10.3390/brainsci10070458

**Published:** 2020-07-17

**Authors:** Marta Huguet-Rodríguez, José Luis Arias-Buría, Belén Huguet-Rodríguez, Rocío Blanco-Barrero, Daniel Braña-Sirgo, Javier Güeita-Rodríguez

**Affiliations:** 1 San José Institute Foundation, aquatic therapy unit, 28054 Madrid, Spain; marta.huguet@ohsjd.es (M.H.-R.); rocio.blanco@ohsjd.es (R.B.-B.); daniel.brana@ohsjd.es (D.B.-S.); 2 Department of Physical Therapy, Occupational Therapy, Rehabilitation and Physical Medicine, Universidad Rey Juan Carlos, Alcorcón, 28922 Madrid, Spain; joseluis.arias@urjc.es; 3 Servicio Pediatria del Hospital Doce de Octubre, 28041 Madrid, Spain; belen.huguet@salud.madrid.org; 4 Research Group of Humanities and Qualitative Research in Health Science of Universidad Rey Juan Carlos (Hum & QRinHS), Universidad Rey Juan Carlos, Alcorcón, 28922 Madrid, Spain

**Keywords:** neuromuscular disorder, children, respiratory function, functional skills, aquatic therapy

## Abstract

Neuromuscular disorders (NMD) lead to the progressive loss of motor and respiratory functions and a decline in daily activities and participation. We aimed to evaluate respiratory changes and functional outcomes in children attending an aquatic therapy program. Eleven patients diagnosed with NMD (4–18 years, Vignos scale 1–9) were involved in a 10-week aquatic exercise program. The ventilation variables were: peak cough flow, volumes (forced expiratory volume in one second-FEV_1_ and inspiratory volume) and respiratory pressures to evaluate strength and oxygen saturation (O_2_ sat). Functional skills were measured in the aquatic environment (Water Orientation Test Alyn 1) and on dry land, (Pediatric Evaluation of Disability Inventory), together with quality of life (Pediatric Quality of Life Inventory). Our evaluation included several 2 × 6 mixed-model repeated measures analysis of covariance (ANCOVA) with time (baseline, post 1 session, pre-post at five weeks and pre-post at 10 weeks). Important improvements in functional skills were observed in and out of the water and children under the age of 11 displayed a significant difference for inspirational volume (*p* = 0.002) and O_2_ sat (*p* = 0.029). Clinical, statistically insignificant changes were found for peak cough flow and expiratory pressures values after aquatic exercise. These results may support a relationship between aquatic exercise in NMD, respiratory outcomes and functional activities in water and on land.

## 1. Introduction

Neuromuscular disorders (NMD) constitute a wide group of pathologies that lead to an alteration of the peripheral nervous system, which can occur in cells of the anterior horn, peripheral nerves, neuromuscular junction and muscle [1,2]. They may begin in childhood or, in adulthood and life expectancy is conditioned by the type of specific disease and degree of involvement [2,3].

NMDs are characterized by proximal weakness, which is usually symmetrical, progressive muscle atrophy and loss of respiratory function. Structural alterations (postural, scoliosis, joint deformities, muscle contractures) and functional alterations (decreased mobility, and significant alteration of respiratory function) are produced by muscle weakness [4,5,6], which also impacts on the control of static and dynamic postural balance [7]. Maintenance of trunk symmetry is important to preserve functional movements and position and to prevent deformities [8], because the muscles of the trunk play a crucial role in the respiratory functions of children with NMD [9]. Thoracic and oropharyngeal muscle weakness makes it difficult to eliminate secretions due to difficulty in coughing. It also results in decreased movement of the chest and expansion of the rib cage during inspiration, decreasing forced vital capacity (FVC) and total lung capacity, as well as forced residual capacity [10,11]. Respiratory function decreases by about 10% per year in patients with Duchenne muscular dystrophy (DMD) [12]. This decline results in stress and cardiopulmonary exercise intolerance [13], contributing to decrease daily activities and participation, and assessment is a simple way to monitor disease progression [14]. The association between timed walking tests and the progression of numerous neuromuscular conditions makes functional activities such as walking an essential, yet simple, means of monitoring and detection [14].

Kamen et al. [15] suggest that resistance exercises can alter the unloading properties of motor units, which represent an important neural mechanism that promotes early and rapid gains in muscle strength capacity. Siddique et al. [16], provided a quantitative scheme of neural adaptations to resistance training. They showed that the neural adaptations that accompany increased muscle strength probably arise from subtle changes throughout the entire neuro-axis, with contributions from cortical and subcortical mechanisms. This meta-analysis revealed that resistance training, compared to non-training, modified cortical and subcortical motor circuits that act globally to improve motor neuron pool activation [16]. Their results further demonstrate that the neuronal adaptations of resistance training probably involve both cortical and subcortical adaptations acting to increase motor neuron activation which, at least in part, support some of the increase in muscle strength related to training. In addition, exercise is a common practice to promote health as secondary prevention in adult and pediatric neurological populations, while minimizing secondary consequences [17]. The effects of exercise on the release of neuroprotective factors, on the synthesis of neurotransmitters or on neurogenesis, initially induced by an increase in cardiac output, are well known [17].

The guidelines developed by the working group on the care of children with NMD state that aquatic exercise should be performed, as they recommend low-impact exercise and strengthening exercises combined with aerobic exercise (running, swimming, walking) as they promote improvements in strength and maintenance of structures, as well as functionality [4,5]. Nonetheless, there is currently no precise information regarding dose, frequency and intensity [5]. Exercise in water is used early in initial phases of many pathologies, as it is a feasible exercise modality that is low-impact and requires low-energy consumption. The aquatic environment provides hydrokinetic and hydrodynamic characteristics that can encourage this type of work [18], causing changes in respiratory mechanics. The body produces an increase in blood pressure, as well as surface pressure in the pleura and compression of the rib cage, causing the diaphragm to rise [18], increasing respiratory frequency and effort. Inspiratory resistance training during immersion with water at neck level, may increase respiratory muscle strength and endurance and result in exercise tolerance, increasing the vital capacity for functional activities such as walking [18].

Despite the relevant effects of aquatic therapy studied in other childhood pathologies [19], information on its application and efficacy in patients with NMD is still very limited. In children with Spinal Muscle Atrophy (SMA), benefits have been evaluated in gait [20], strength [21], and maintenance of functional activity [22]. In children with DMD, improvements in functional abilities have been found [23] despite the lack of a standardized treatment protocol. At the respiratory level Voos et al. [9] investigated the evolution of timed immersion expiration, and its relationship with age, motor and respiratory functions in patients with muscular dystrophies. However, no previous study has investigated the impact of aquatic exercise on respiratory functions and functional activities in water and on dry land. Our hypothesis is that aquatic exercise optimizes respiratory functions, allowing children with NMD to participate more in their daily lives. The aim of this study was to evaluate the respiratory changes in children with NMD participating in an aquatic therapy program. As a secondary objective, we evaluated functional outcomes and quality of life.

## 2. Materials and Methods

### 2.1. Participants

The target population for this study were patients who presented a pathology within the spectrum of neuromuscular diseases. The patients were selected directly from those who were actively undergoing treatment at the water therapy unit of the Fundación Instituto San José, in Madrid, Spain.

The study participants had to meet the following inclusion criteria: (a) age ≥4 years and ≤18 years (the American Thoracic Society (ATS) advises that the first evaluations be performed around 4–6 years [24]); (b) diagnosis of neuromuscular disease based on molecular genetic analysis, (c) receiving continued treatment with aquatic therapy, for at least three months prior to the start of the study because prescribed techniques need time to produce solid physiological and structural changes; (d) sufficient capacity to understand simple requests and signing of the informed consent by the legal guardians.

**Figure 1:**
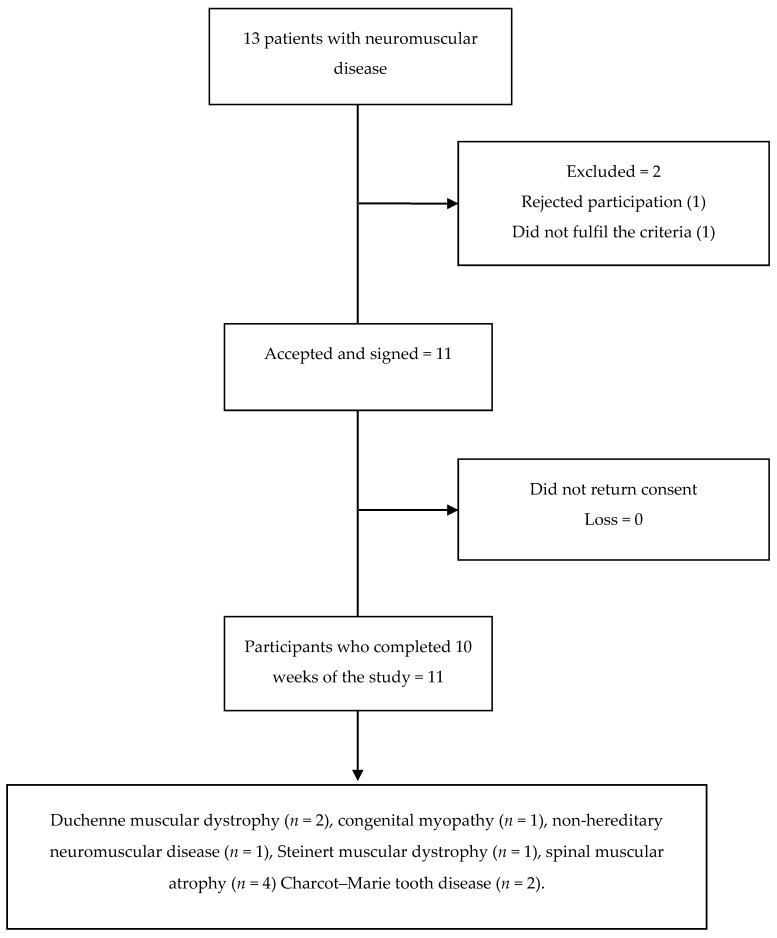
Flow diagram.

The exclusion criteria were: (a) coexistence of other chronic debilitating syndromes; (b) coexistence of other concomitant diseases that could affect the respiratory level; (c) being subject to pharmacological intervention for acute respiratory pathology (not being adapted to the doses of corticoids in patients with NMD); (d) not reaching a peak cough flow (PCF) of 60 L/min; (e) not being able to perform an oral occlusion around a mouthpiece used for diving or a conventional type of mouthpiece; (f) lack of informed consent by the patient/legal guardian and/or patient’s request to be excluded at any time from the study. The reasons for exclusion can be found in Figure 1, which provides a flowchart of patient recruitment and retention.

Throughout the study period, all participants continued their weekly sessions of physical therapy, occupational therapy and adapted sports on dry land, with an average of three sessions per week and a duration of 45–60 min.

### 2.2. Data Collection

Intra-study measurements were made by the same researcher, who had no previous contact with any of the participants. Codes were assigned to participants who were known only to one researcher in the group who neither performed the intervention nor the scans. The assessment was performed on the day of the aquatic therapy session, before and after the same. First, the Borg scale (visual) was performed, subsequently the oxygen saturation (O_2_ sat) and heart rate (HR) were measured, followed by the volume of inhaled air, PCF, forced expiratory volume in one second (FEV_1_) and the strength of the respiratory muscles.

#### Outcome Measures

To measure the PCF, a peak flow meter was used (Datospir Peak 10, Sibelmed, Spain). Obtaining a measurement in L/min, this measures determines the patient’s ability to perform a productive cough and is of great clinical importance [25,26]. To obtain the PCF, the child is asked to take a deep breath and then cough as hard as possible. Three attempts are made, discarding the most disparate and averaging the remaining two [24,27].

A digital lung function meter (Vitalograph COPD-6, Ireland) was used to measure the volume of exhaled air by means of a forced expiratory volume in one second-FEV_1_ and in six seconds (FEV_6_). The Vitalograph COPD-6 meter allows us to obtain measurements of several spirometry values such as the FEV_1_ as well as the ratio between FEV_1_ and FEV_6_, which is related to FVC. Previous studies have shown that this device has 95% validity for obtaining FEV_1_ and the FEV_1_/FEV_6_ ratio [28].

To measure the strength of the respiratory muscles, the Maximal static inspiratory (Pi_max_) and expiratory (Pe_max_) pressures were measured, using a respiratory pressure meter (Micro RPM, Hoechberg, Germany). Measurement of muscle strength is considered an important value in determining respiratory capabilities in children with neuromuscular disease [29]. To determine this capacity, one of the most commonly used methods is to obtain the values for maximum expiratory pressure value (Pe_max_) and maximum inspiratory pressure (Pi_max_,) [27,29,30].

To measure the volume of inhaled air, a volumetric stimulator was used (AirLife volumetric incentive spirometer, Vyaire Medical, Illinois, USA). This is a tool used for breathing exercises, although it allows us to know the volume of air that the child is capable of inhaling.

As safety measures before and after water exercise, the measurement of O_2_ Sat and HR were evaluated using a pulse oximeter (fingertip Oximeter, Creative Medical, Qingdao, China) and the perceived exertion scale for children (EPinfant), developed according to the Borg scale. However, the intensity of the dosage of aquatic therapy exercises was not controlled during the sessions.

To evaluate the child’s ability to adjust to the aquatic environment and related functional skill, the Water Orientation Test Alyn (WOTA) 1 was used. This test consists of 15 items scored from 0 to 3 depending on the skill of performing the functional task in the aquatic environment. The WOTA 1 scale is reliable and valid for assessing mental adjustment and aquatic function in children with disabilities [31]. The test–retest reliability for total score has been found to be excellent for WOTA1 (Intraclass Correlation Coefficient = 0.97).

The pediatric disability inventory (PEDI) scale was used to measure functional skills and ability to perform activities of daily living. It is subdivided into three domains: self-care (73 items), mobility (55 items), and social function (65 items), and consists of three distinct sections that include these domains; functional skills, caregiver assistance and adaptations. For the present study, only the functional skills section was used. This scale has good sensitivity to change and high reliability, both in terms of internal consistency and reproducibility, whether intra-class, test-retest or inter-interview [32,33].

To measure the quality of life of children with neuromuscular disease, the pediatric quality of life inventory (PedsQL) was used, employing the version for children and adolescents (2–18 years) with neuromuscular disease. The scale is divided into three dimensions; “about neuromuscular disease” (17 questions), “communication” (3 questions) and “about our family resources” (5 questions). Except for the version for the 5–7-year-old age group, only the parent questionnaire has these dimensions, whereas the questionnaire for children only evaluates the “about neuromuscular disease” dimension [33,34]. This scale has excellent psychometric properties of validity, reliability and external consistency in its validation and translation into Spanish [33,34].

### 2.3. Procedure

An aquatic therapy program was developed consisting of one 45 min session per week at the Unidad de Terapia en el Agua de la Fundación Instituto San José. (Table S1. Intervention program). The physical therapists who conducted the intervention were specialists in aquatic therapy, with an average of three years of experience in the pediatric neuromuscular field under study.

### 2.4. Statistical Analysis

The statistical analysis of the data from the outcome measures was performed using IBM SPSS Statistics (version 21, Armonk, NY, USA). A value of *p* < 0.05 was established to determine statistical significance.

A descriptive analysis of the entire sample was performed. Data for quantitative variables were described using trend measures (mean (Mo) and standard deviation (SD)), whereas qualitative variables were described using frequencies (%). In order to know the inter-group evolution, comparisons were made using the ANOVA statistical test of 6 × 2 repeated measurements, with six measurement moments (PRE/POST during the 1st session + PRE/POST at 5 sessions + PRE/POST at 10 sessions) and with 2 different recoded groups: age variable (young (4 years ≤ age <11 years) vs. older (11≤ age ≤18 years)) and the sex variable (girl vs. boy).

### 2.5. Ethical Considerations

This study was approved by the Research Committee of Fundación Instituto San José (Code IP08) and by the Ethics Committee of the Universidad CEU-San Pablo (approval code: 266/19/TFM). Furthermore, this study adhered to the principles stated in the WMA Declaration of Helsinki [35]. Written consent and permission to record the interviews were obtained from all of participants and their legal guardians in the case of underage participants.

## 3. Results

### Descriptive Characteristics of the Sample

The sample consisted of 11 participants, 5 girls (45.45%) and 6 boys (54.54%), aged 4 to 18 years. The mean age of the study sample was 8.36 (SD ± 4.43) years. The most predominant motor function level (Vignos scale) was 9 (27.27%), which represented children using self-propelled wheelchairs for transportation. Table 1 displays the independent baseline variables of the participants. None of the sessions had to be interrupted for safety reasons and none of the children reported adverse effects during the sessions.

**Figure 2:**
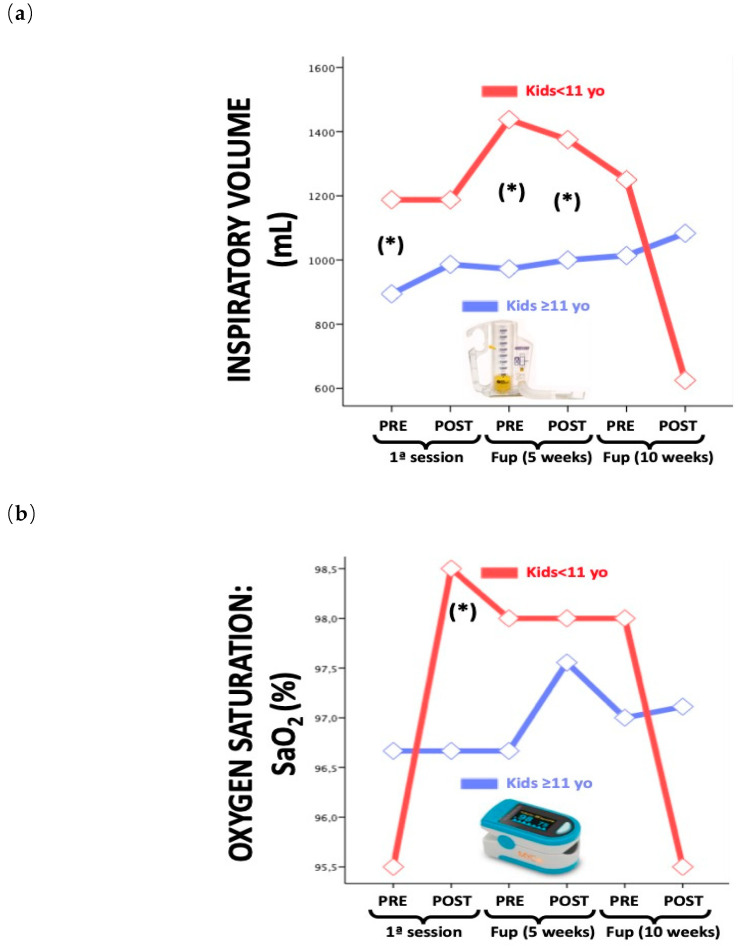
Respiratory variables by age. (**a**); Inspiratory volume (**b**) O_2_ saturation. * = *p* < 0.05

We found significant differences in inspirational volume (*p* = 0.002) and O_2_ sat (*p* = 0.029) for children under the age of 11. (Figure 2a,b)

Clinical, statistically insignificant changes were found for peak cough flow and expiratory pressures after aquatic exercise (Table 2).

Although not significant, important improvements in functional skills were observed both in and out of the water. The WOTA 1 scale increased and improved by almost 3.37 points. The PEDI scale improved for all dimensions, both the self-care dimension (increasing by 3.37 points), the mobility dimension (increasing by 3 points) and the social function dimension (increasing by 1.45 points). The PedsQL questionnaire indicated improvement in its three dimensions, both in the disease (increasing by 0.99 points), communication (increasing by 4.17 points) and family functioning (increasing by 2.86 points) (Table 3).

## 4. Discussion

The present research was carried out to evaluate the changes that may occur in respiratory function in children with NMD, after intervention based on aquatic exercise and its influence on subsequent functional abilities, both in the aquatic environment and on dry land and the impact of this intervention on quality of life. Improvements in the performance of functional mobility tasks in and out of the water were found, together with improvements in inspiratory and expiratory volumes, which could indicate a possible relationship between aquatic exercise in NMD and respiratory functions related to functional participation.

Our results show an increase in PCF values according to our measurements. The PCF variable represents a high clinical relevance and is considered an indicator of health status in patients with NMD, since it reflects the expiratory flow thus monitoring expiratory muscle weakness through coughing. The ability to produce a good cough also depends on the capacity to perform a full pre-cough inhalation, which is in line with our hypothesis regarding the increased inhalation that occurs in the aquatic environment due to the pressure generated upon the thoracic cage thanks to the hydrostatic pressure of the water, which leads to an increase in the PCF. The natural tendency in patients with NMD is a decrease in PCF, due to muscle weakness, and therefore we interpret the increase in our short-term results in PCF as being clinically significant, in line with results from Voos et al. who found improvements in timed immersion mouth expiration and timed immersion nose expiration [9]. However, this prior study found no change in peak expiratory flow and FVC, both measured using spirometry. For these authors, the control of expiration in water immersion may diverge from spirometry, since they found that some patients may show a deterioration of respiratory function in spirometry, yet good mouth and nose expiration times in immersion. Hydrostatic pressure helps to stabilize the trunk by activating the abdominal muscles, because the chest is immersed [18]. Therefore, the diaphragm becomes more efficient and this may explain the improved breathing control in the pool. This seems to corroborate the fact that various compensatory strategies for movement and posture are available in the aquatic environment [9]. Hind also proposed that trunk control is optimized in children with DMD in the water, due to the hydrostatic pressure, which also improves body perception and even proprioception, according to this author’s proposals for research protocols in the aquatic environment [23].

In spite of not finding statistically significant changes in our sample, we observed a significant clinical increase based on the assessments of the Pe_max_ variable, and a decrease of the Pi_max_ during the 10-week follow-up. This improvement in pressure is most likely influenced by the strength exerted by children during inspiration and expiration, probably due to the muscle training that takes place during the aquatic therapy sessions. The effects on the inspiratory muscles is considered particularly striking as greater lung insufflation is provoked, together with thoracic movement providing the body with increased stability. Similarly, outside the water, Zeren et al. showed an increase in the Pi_max_ and Pe_max_, during inspiratory muscle training, as an independent predictor for the limits of postural stability in children with chronic lung diseases, who increased their postural scores [36]. The inspiratory training in our study is facilitated by the pressure that the water generates on the chest of the patients, offering resistance to inspiration during movement in the water, necessary for the training of the involved muscles. The increase found in Pi_max_, at the end of our intervention could indicate that, by performing aquatic exercise, the increase in the use of the inspiratory musculature is stimulated, resulting in improvements affecting both posture and movement.

We believe that the differences in respiratory variables in relation to age are most likely due to the fact that children under 11 years of age do not yet have a mature respiratory system that is responsive to physical therapy treatment, nor are they sufficiently aware of optimal gesture performance (neither during aquatic exercise nor concerning the correct use of the inspirometer with correct mouthpiece sealing).

In this study, the values of O_2_ sat, HR and perceived effort were included as safety parameters and measures of the patients’ cardiovascular condition. There was an increase in O_2_ sat after aquatic exercise and a decrease in HR. We believe that this has an effect on respiratory capacity since a decrease in HR and an increase in O_2_ sat, could imply that an increase in gas exchange, favorable to blood oxygenation is taking place, decreasing work at the cardiovascular level. In addition, the children’s perceived effort has always been favorable with their values decreasing, both after the first session and at the end of the 10-week aquatic program. This is probably due to the fact that the aquatic environment promotes the maintenance of active movement and, consequently, greater independence for a longer period. Despite this, the child’s perceived effort during aquatic exercise should be taken into account, bearing in mind that this is dependent on each subject’s own understanding.

Regarding the functional skills both inside the water (WOTA 1) and on dry land (PEDI) the mean values obtained progressively increased in each of the measurements. Concerning the values obtained in the WOTA 1, a progressive increase in the score was observed, increasing between the first and last measurement. In addition, in the PEDI, an increase was observed for all dimensions, including the self-care dimension, mobility and social function. In our opinion, functional skills did not significantly improve in this aquatic intervention due to the natural evolution of the condition. This creates a directly proportional relationship in children with their developmental growth. Often, both are so rapid that it makes it difficult for our patients to adapt and generate functional skills, because when they do, their status changes once again. All this—added to the fact that, in spite of being an individualized therapy—not all children respond equally to the same protocol of aquatic exercises due to the evolution of the process of maturity. Thus, in children with a larger physical constitution it would be more necessary to increase the working time or even the resistance which we are able to generate with accessories to increase the workload.

In addition, the quality of life scores, using the PedsQL scale in its version for NMD, did not reveal significant changes; an increase in the mean results was only found in the module for parents, regarding the disease. This may be due to the short duration of our intervention program, as a 10-week period may not be sufficiently sensitive for registering perceived changes in quality of life, as this is a relatively broad variable. Similar findings were found by Montes et al. [37], who investigated the influence of physical activity, combining a program of strength training and aerobic exercise, regarding usual care, in patients with SMA type III, during six months. In their study they found that regarding quality of life in patients with SMA, no statistically significant changes were observed in PedsQL values, in line with our findings.

However, the clinical impact of the findings found in the present study could be considered noteworthy, since patients with neuromuscular pathology generally tend to have progressive muscle weakness, which leads to an alteration in their capacities and functionality. Therefore, it is clinically relevant that they maintain and even improve some parameters in the short term in relation to the variables studied, through the performance of aquatic therapy. However, this study had several limitations. First, the lack of a control group. The patients in this study have maintained their normal activity in the dry land environment, performing their usual therapies, therefore we cannot consider that the changes are motivated only by the aquatic therapy. Second, concerning the ventilatory variables, only the capacity to cough and the respiratory muscle strength were taken into account as being important and representative variables in relation to the respiratory capacities of children with NMD. However, it would have been interesting to determine FVC via spirometry. This was not performed due to the discrepancies in the feasibility of the test in preschool age. Finally, the intensity of aquatic exercise was not controlled, as a result, the fatigue component that occurs due to weakness in NMD was not taken into account.

Therefore, the results of this study should be treated with caution and the design of robust studies with a control group is suggested as the main lines of research that emerge from this study. Likewise, we suggest increasing the sample size of patients with NMD, to increase the statistical power of findings and to enable extrapolation of findings to the wider study population. A long-term follow-up of the changes produced in patients with NMD should also be considered in future research, as well as evaluating the implication and relevance of these changes on the daily lives of children with NMD and their families.

## 5. Conclusions

An aquatic exercise program in children with NMD appears to promote short-term increases in certain clinically relevant concepts, such as PCF, inspiratory volume and ventilatory pressures, described as indicators of health status and progression in these pathologies. An increase in O_2_ saturation after the interventions and a decrease in HR were found, as measures of patients’ cardiovascular status.

Likewise, increases were shown in values measuring functional skills both inside (WOTA 1) and outside water (PEDI), for the domains of self-care, mobility and social function. These results may support a relationship between aquatic exercise in NMD and functional participation-related respiratory functions, suggesting that resistance exercises may alter the unloading properties of motor units, representing an important neural mechanism that mediates early and rapid gains in muscle strength capacity with influence on daily life. Further studies are certainly needed to assess the extent of efficacy of aquatic therapy in NMD disorders: to this regard it would be useful to perform in the future case–control study having as changing variable the aquatic therapy comparing two groups of SMA patients equally receiving nusinersen therapy.

## Figures and Tables

**Table 1:** Participants independent baseline variables.

Participant Code	Diagnosis of the Child	Age (Years)	Sex	Height (cm)	Weight (kg)	Time Since Began Aquatic Therapy (Months)	Vignos Scale	Mechanical Ventilation
S1	DMD	18	M	157	61	48	7	Does not require
S2	Congenital myopathy	7	M	129	23	18	2	Does not require
S3	NMD non hereditary	10	M	142	32	18	1	Does not require
S4	SMA type II	5	M	115	17	24	9	Nocturnal BiPAP
S5	SMA type II	4	M	100	15.6	15	9	Nocturnal BiPAP
S6	SMA type III	5	F	107	15	20	2	Does not require
S7	DMD	11	M	146	63	21	9	Does not require
S8	SMA type II	4	F	98	12	18	8	Does not require
S9	CMT	5	F	119	20	24	3	Does not require
S10	CMT	12	F	150	45	24	7	Does not require
S11	Steinert myotonic dystrophy	11	F	152	40	6	1	Does not require

DMD—Duchenne muscular dystrophy; NMD—neuromuscular disorder; SMA—spinal muscular atrophy; CMT—Charcot–Marie-Tooth; BiPAP—bilevel positive airway pressure.

**Table 2:** Values of respiratory variables.

Measurement Moments	1st Session of Aquatic Therapy		5th Session of AT		10th Session of AT		*p*-Value
Mean/CI Outcome Measures	PRE: Baseline	POST	PRE	POST	PRE	POST: Final	
FEV_1_ Mean CI	1.3 ± 0.61 0.4–2.14	1.14 ± 0.52 0.4–1.99	1.12 ± 0.48 0.5–2.16	1.23 ± 0.53 0.52–2.02	1.04 ± 0.58 0.38–2.09	1.22 ± 0.59 0.45–2.13	>0.05
PCF Mean CI	156.36 ± 63.45 60–250	160.91 ± 61.23 70–250	164.55 ± 75.28 70–270	166.36 ± 73.39 70–260	169.09 ± 78.54 70–270	174.55 ± 76.07 70–270	>0.05
MEP Mean CI	33.91 ± 17.64 8–57	33.18 ± 20.05 7–66	33.36 ± 17.41 6–54	33.09 ± 15.68 11–53	35.45 ± 18.09 11–56	37.82 ± 19.66 12–63	>0.05
MIP Mean CI	−27.18 ±16.28 −50–−4	−30.36 ± 18.51 −57–−1	−31.64 ± 14.62 −48–−7	-34.27 ± 19.08 −59–−9	−34.64 ± 14.3 −52–−9	−35.82 ± 19.67 −59–−12	>0.05
Inspired VOL Mean CI	947.73 ± 469.5 200–1.750	1.022.73 ± 446.58 250–2.000	1.056.82 ± 441.46 375–1.750	1.137.50 ± 505.01 250–2.000	1.056.82 ± 560.03 250–2.125	1.000.00 ± 594.24 125–2.375	0.002
O_2_ SAT Mean CI	96.45 ± 1.44 94–98	97 ± 1.55 94–99	96.91 ± 1.3 95–98	97.64 ± 0.67 97–99	97.18 ± 0.75 96–98	96.82 ± 1.17 95–99	0.029

FEV_1_: forced expiratory volume in one second; PCF: peak cough flow; MEP: Maximum expiratory pressure; MIP: Maximum inspiratory pressure; Inspired Vol: Inspired volume; O_2_ sat: Oxygen saturation; CI: Confidence interval.

**Table 3:** Outcome measurement values.

Measurement Moments	Baseline: 1st Session of Aquatic Therapy				Follow-up: 5th Session of AP				Follow-up: 10th Session of AP				*p*
Outcome Measures	MEAN 1	DS 1	MIN 1	MAX 1	MEAN 2	DS 2	MIN 2	MAX 2	MEAN 3	DS 3	MIN 3	MAX 3	
WOTA 1	42.36	8.2	28	50	43.09	7.45	30	50	45.73	5.55	34	51	>0.05
PEDI													
Selfcare	51.36	11.52	35	68	52.36	11.47	38	69	54.73	10.36	41	70	>0.05
Mobility	28.55	18.37	8	54	30.55	19.05	9	53	31.55	18.34	10	52	>0.05
Social function	58.64	4.2	53	65	59.45	3.98	53	65	60.09	4.11	54	65	>0.05
PEDSQL													
D (p)	70.26	13.39	54.41	94.11	71.68	10.31	55.89	90	71.25	7.54	58.8	82.35	>0.05
C (p)	64.77	38.34	25.1	100	63.63	33.6	27.99	100	68.94	30.75	16.67	100	>0.05
FF (p)	66.5	14.92	45	90	67.73	20.17	35	90	63.64	18.99	25	95	>0.05
TOTAL p	70.73	12.92	51	98	69.55	11.94	50	88.04	66.6	6.01	56	74	>0.05
TOTAL c	79.52	9.4	65	94.71	75.68	6.99	61	82.35	73.65	8.39	58	87	>0.05

D—disease; C—communication; FF—family functioning; p—parents; c—children.

## Supplementary Materials

The following are available online at https://www.mdpi.com/2076-3425/10/7/458/s1, Table S1: Intervention program.
