# Supplementary material for: Impact of Aquatic Exercise on Respiratory Outcomes and Functional Activities in Children with Neuromuscular Disorders: Findings from an Open-Label and Prospective Preliminary Pilot Study
Source: Brain Sci. 2020 Jul 17;10(7):458. doi: 10.3390/brainsci10070458 (PMC7407397; doi:10.3390/brainsci10070458)
Supplement: Supplementary file 1 [file brainsci-10-00458-s001.pdf]

Supplementary Material.

Table S1. Intervention Program.

Aquatic Therapy Goals	Treatment Phase	Content	Health Domain	Duration
Mental adjustment + Development of water skills and swimming patterns	Initial phase	Activities that are part of the entrance ritual, are carried out, adjustment to the physical and psychological aquatic environment.<br>Autonomous movement in the environment is encouraged, in dorsal and/or ventral flotation.<br>Free movement<br>▪ With variations in assistance; Flotation aids<br>▪ Control or handling by the therapist	Functions and Structure Activity Participation	10′
Rotational axis WST- Halliwick	Central phase	Rotational axis control is worked on; transversal, sagittal, longitudinal:<br>▪ Flotation aids<br>▪ Handling or assistance provided by therapist<br>▪ Total patient autonomy<br>Loading exercises are performed, according to the motor skills and specific goals;<br>▪ sitting<br>▪ standing<br>▪ all fours<br>▪ kneeling	Functions and Structure Activity	10′
Loading activities + specific mobility and motor skills work	Central phase	Body weight (%) at different levels of immersion.<br>- Xiphoid Appendix (30%)<br>-Pelvis (60%)<br><br>Activity that involves balance control and proprioception, with increased difficulty (using obstacles, different heights, materials that make activity difficult and single leg standing)<br>This includes activities involving; Double tasking<br>▪ Coordination UL-LL<br>▪ Cognitive-motor<br>▪ Dissociation of shoulder and pelvic girdles<br>▪ Flexibility<br>Specific activity for maintenance of ROM	Functions and structure Activity	15′

Mental inversion<br>+<br>Improvement of apneas	Final phase	Immersion activities are carried out. Variations in the level of immersion depending on the capabilities and control of the therapist<br>Group play, putting things away.	Functions and structure<br>Activity<br>Participation	10′

ROM: Range of motion; UL: upper limbs; LL: lower limbs; WST: Water Specific Therapy.
